# Merkel Cell Carcinoma Analysis of Outcomes: A 30-Year Experience

**DOI:** 10.1371/journal.pone.0129476

**Published:** 2015-06-08

**Authors:** Evan Liang, Jeffrey V. Brower, Stephanie R. Rice, Darya G. Buehler, Sandeep Saha, Randall J. Kimple

**Affiliations:** 1 Department of Human Oncology, University of Wisconsin School of Medicine and Public Health, University of Wisconsin—Madison, Madison, WI, United States of America; 2 Department of Pathology, University of Wisconsin School of Medicine and Public Health, University of Wisconsin—Madison, Madison, WI, United States of America; 3 Department of Biostatistics, University of Wisconsin School of Medicine and Public Health, University of Wisconsin—Madison, Madison, WI, United States of America; 4 University of Wisconsin Carbone Cancer Center, University of Wisconsin School of Medicine and Public Health, University of Wisconsin—Madison, Madison, WI, United States of America; School of Medicine, Fu Jen Catholic University, TAIWAN

## Abstract

**Background:**

Merkel cell carcinoma (MCC) is an aggressive cutaneous malignancy with poor prognosis. Limited data exists to guide treatment decisions. Here we report on our institutional experience and outcomes treating patients with MCC.

**Methods:**

A database search (1984-2014) of patients treated at the University of Wisconsin Hospital and Clinics was used to identify patients with histologically confirmed MCC. Patient, tumor, and treatment characteristics were examined via review of medical records. Statistical analyses were performed to assess outcomes and associated prognostic factors.

**Results:**

A total of 87 patients with MCC were identified with a median follow-up of 17 months (mean: 38, range: 0-210 months). Two and five-year overall survival rates were 53.9% and 32.8%, respectively. Recurrence was documented in 31.0% of patients (85.2% locoregional, 48.1% distant and 33.3% both). Patients with a history of immunosuppression exhibited significantly worse survival (hazard ratio, 2.01; 95% CI, 1.1-3.7) when compared to immune-competent individuals. The head and neck region was the most common location of primary lesion (N=49) followed by the extremities (N=31). Upper extremity primaries predicted significantly better overall survival (hazard ratio, 0.48; 95% CI, 0.23-0.99) while lower extremity primaries did not have significantly better results (hazard ratio, 0.5; 95% CI, 0.21-1.2) in comparison to head and neck site of primary. Nodal involvement (hazard ratio, 2.95; 95% CI, 1.5-5.79) was also a negative prognostic factor associated with poor overall survival when compared with clinically node negative patients. Primary tumor size > 2 cm (hazard ratio, 1.76; 95% CI, 0.91-3.4) was not associated with survival.

**Conclusions:**

This study highlights the role of various factors in determining prognosis of Merkel cell carcinoma; history of immunosuppression, nodal involvement, and head/neck primary predicted worse overall survival. These findings suggest that improvements in both distant and locoregionally directed therapies might play an important role in control of MCC and identify areas for future study.

## Introduction

Merkel Cell Carcinoma (MCC) is an aggressive skin cancer with a 5-year overall survival rate of approximately 50% [1]. Fortunately, MCC is a rare malignancy with an estimated incidence of 0.32 cases per 100,000 person-years (about 800 cases/year) in the United States [2, 3]. MCC was first characterized in 1972 as “trabecular carcinoma of the skin” [4, 5]. The malignancy was subsequently renamed after the presumed cells of origin when further investigation suggested that the cancer originated in the neuroendocrine Merkel cells within the basal layer of the epidermis [6]. Benign Merkel cells in the skin have more recently been noted to lack proliferative capacity, and it is now suspected that MCC does not develop from differentiated Merkel cells but rather from cutaneous progenitor cells [7]. The incidence of MCC has risen since it was first described in the 1980s due to both improved diagnosis and increased exposure to known risk factors such as UV exposure and chronic immunosuppression [8–10]. MCC is most commonly seen in Caucasian men, and while they can arise in any area of the skin, they are most common in the head and neck region [2]. This relatively rare malignancy is of particular scientific interest due to its correlation with Merkel Cell Polyomavirus infection, as first described in 2008 [11]. Since then, studies have looked into the clinical impact of polyomavirus seropositivity [12–17], as well as that of cell cycle proteins such as c-KIT [18, 19], Bcl-2 [20, 21], p53 [18, 22], and p63 [22–25].

The failure pattern for MCC can involve local recurrences and/or the development of regional nodal or distant metastases. Due to the rarity of the disease, no prospective studies have been or are likely to be performed to determine the optimal treatment regimen. Several groups have reported institutional experience treating MCC[26–35] and large surveys like Surveillance, Epidemiology, and End Results Program (SEER) analyses have been recently published [1, 36–39]. Factors shown to be predictive of survival in these studies include tumor stage, immunosuppression status, lymph node involvement and male sex [32, 34, 40–43]. Merkel cell is also of particular interest because of a potential viral etiology through Merkel cell polyomavirus [11].

Given the rarity of this disease, and its relatively recent recognition, the optimal treatment remains uncertain [2, 42]. While most groups recommend maximal safe surgical excision, the effectiveness of adjuvant therapies, the cases in which they should be utilized, and the optimal regimens remains unclear [36, 37, 44–47]. We sought to add to the published literature by reporting our experience with patients treated for MCC at our tertiary care referral center over the last 30 years. This retrospective clinical study characterizes a cohort of patients with pathologically confirmed MCC with a description of the clinical presentation, treatments rendered and outcomes.

## Methods

### Patients

Approval for this study was obtained from the University of Wisconsin Health Sciences Institutional Review Board. No consent was required due to the retrospective nature of this study. All data was analyzed anonymously. Study design adhered to the guidelines put forth by the Strengthening the Reporting of Observational studies in Epidemiology initiative (STROBE, www.strobe-statement.org). Patients with MCC diagnosed between January 1984 and June 2014 were identified using the PowerPath Surgical Pathology database (Sunquest Information Systems, Tucson, AZ). This search identified both patients originally diagnosed with MCC at our institution and those whose case was reviewed at UW as part of a second opinion. Those seen at our institution had diagnoses confirmed by immunohistochemistry—cytokeratin 20 and CAM5.2 positivity coupled with CD45 negativity. Medical records were abstracted to determine relevant past medical history, tumor characteristics at presentation, history of the treatments rendered for MCC, and cancer-specific follow-up data.

Information was gathered about tobacco use, previous cancer diagnoses, coronary artery disease, chronic obstructive pulmonary disease, type 2 diabetes mellitus, and immunosuppression. Immunosuppression status was considered positive if the patients had any history of solid organ transplant, were taking chronic immunosuppressive drugs, or had a prior or current diagnosis of chronic leukemia or lymphoma. Patients were restaged with available data following the guidelines of the American Joint Committee on Cancer’s Cancer Staging Handbook, seventh edition [48]. Tumor staging was determined following surgical excision of the primary tumor, while nodal staging for the purposes of this study was determined at the time of the most detailed nodal evaluation (physical exam, imaging, sentinel lymph node biopsy, or lymph node dissection) at the time of initial disease presentation. Treatment variables gathered included the excisional surgical procedure performed, nodal evaluation method, and course of adjuvant therapy provided. Treatments were excluded from analysis if they were administered following a confirmed recurrence of the disease.

Endpoint events including the first recurrence after the initial treatment, first locoregional recurrence, local recurrence, as well as death were abstracted from the medical records. Dates of death, when unavailable, were obtained from the Social Security Death Index. Endpoint survival times were measured from the date of confirmed histological diagnosis of MCC, including the histological diagnoses made outside our institution. Patients who were lost to follow-up before reaching the endpoint in question were censored at the time of their last available follow-up.

### Statistical Analysis

The statistical analyses were performed with the goal of studying the outcomes of patients with Merkel cell carcinoma. Overall survival times for the patients were analyzed using the Kaplan Meier method and summarized using median survival times and 95% confidence interval (95% CI). Hazard ratios and 95% CI were obtained via univariate Cox Proportional Hazards Model (CPHM). A multivariable CPHM was considered to identify and evaluate important variables associated with overall survival in MCC patients using the statistical method of best subset selection according to the Akaike information criterion (AIC)[49]. However, the multivariable analysis was not performed due to high levels of missing data in the subset of the variables of interest. Statistical significance was set at P<.05, and all tests were 2-tailed. All analyses were performed using SAS version 9.4 (SAS Institute, Cary, NC) and GraphPad Prism version 6 (GraphPad Software Inc., La Jolla, CA).

## Results

### Patient Demographic / Past Medical History

Eighty-seven patients were identified between January 1, 1984 and June 30, 2014. Patient, and tumor characteristics for all patients are presented in Table 1. As of September 1, 2014, the median follow-up time was 17 months (mean 38; range: 0–210 months) and 59 deaths were recorded (67.8%). The cohort had a slight male predominance (56.3% vs. 43.7%) with a median age at diagnosis of 75 years (range 44 to 90). Of the 80 patients with a recorded race, the majority (96%) were of European descent, while the remainder were Hispanic (4%), representative of the characteristics of the institutional catchment basin. Other factors included history of smoking (44.9%), chronic immunosuppression (23.8%), and other malignancies (51.2%) (S1 Table). Four (4.6%) patients had a history of hematolymphoid malignancy that was designated as positive for both immunosuppression and prior malignancy. Patients were excluded from the cohort if their status was unknown; 7 patients had unknown smoking status while 3 had unknown past history of immunosuppression or cancer.

**Table 1:** Patient, tumor, and treatment characteristics.

Patient Characteristics	N (% total)	Tumor Characteristics	N (% total)
Age at diagnosis, y (N = 87)		Site of Primary (N = 87)	
Median (range)	75 (44–90)	Head and neck	49 (56.3)
Sex (N = 87)		Upper extremity	19 (21.8)
Male	49 (56.3)	Lower extremity	12 (13.8)
Female	38 (43.7)	Trunk	2 (2.3)
Vital Status (N = 87)		Genitals	1 (1.1)
Alive	28 (32.2)	Unknown (nodal at presentation)	4 (4.6)
Dead	59 (67.8)	T stage—primary tumor (N = 69)	
Race (N = 80)		T0 (no evidence of primary)	4 (5.8)
White	77 (96.3)	T1 (≤2 cm)	46 (66.7)
Hispanic	3 (3.8)	T2 (>2 cm, ≤5 cm)	7 (10.1)
History of Smoking (N = 78)		T3 (>5 cm)	6 (8.7)
No	43 (55.1)	T4 (invasion of bone, muscle, or cartilage)	6 (8.7)
Yes	35 (44.9)	N stage—regional lymph nodes (N = 74)	
History of CAD (N = 84)		cN0 (negative nodes by clinical exam)	27 (25.3)
No	62 (73.8)	pN0 (negative nodes by pathologic exam)	18 (19.5)
Yes	22 (26.2)	N1a (micrometastasis)	6 (6.9)
History of COPD (N = 84)		N1b (macrometastasis)	20 (17.2)
No	77 (91.7)	N2 (in-transit metastasis)	3 (3.4)
Yes	7 (8.3)	M stage—distant metastases (N = 63)	
History of DMII (N = 84)		M0 (no distant metastases)	59 (93.7)
No	64 (76.2)	M1 (distant metastases)	4 (6.3)
Yes	20 (23.8)		
Immunosuppression status (N = 84)			
No	64 (76.1)		
Yes	20 (23.8)		
Prior Cancer (N = 84)			
No	41 (48.8)		
Yes	43 (51.2)		

### Tumor Characteristics at Presentation

Approximately half (56%) of all patients presented with a primary cutaneous tumor of the head and neck (Table 1). The majority of remaining patients had primary cutaneous lesions localized to the upper extremities (21.8%), lower extremities (13.8%), while few patients had lesions involving other sites (8%) such as the trunk or genitals. Four patients (5.8%) presented with lymph node involvement but without a discernable primary site of disease and were characterized as unknown primary. Of the primary cutaneous tumors with staging data available, the majority were 2 cm or less in greatest dimension (T1, 66.7%). Primary lesions between 2 and 5 cm (T2, 10.1%), greater than 5 cm (T3, 8.7%), and those with bone, muscle, or cartilage invasion (T4, 8.7%) were less common. Node negative patients, whether by pathological (pN0, 24.3%) or clinical findings (cN0, 36.5%) were more common than those who were node positive (N1a, N1b, N2; 39.2%), and only a small number of patients presented with distant metastases (M1, 6.3%, n = 63).

### Clinical Management

The majority of patients underwent surgical resection of their primary tumor (Table 2). Two patients were treated with radiotherapy alone, one patient with chemotherapy alone, and two with definitive chemoradiotherapy. Treatment data was not available for five patients. Among those who initially underwent surgical interventions, wide local excision was the most common procedure performed (53.7%). Mohs dermatologic excision (28.0%) was also common in this series, while a number of patients underwent excisional biopsy alone (8.5%). Margin status was available for 58/81 patients (71.6%). Of those, 50 patients had a negative resection margin, 3 patients had close margin (<1 mm) and 5 patients had an incomplete excision. Surgical nodal evaluations were performed for 40 patients; 18 (45.0%) sentinel lymph node biopsies (SLNB), 16 (40.0%) nodal dissections (ND), and 6 (15.0%) cases where both were performed. Of the 77 patients that received initial surgical treatment, 37 (48.1%) received adjuvant radiotherapy, 28 (36.4%) had no adjuvant treatment, and 12 (15.6%) were lost to follow-up without documentation of adjuvant therapy.

**Table 2:** Patient management characteristics.

Primary surgical procedure (N = 82)	
No surgical procedure performed	5 (6.1)
Excisional biopsy only	7 (8.5)
Wide local excision	44 (53.7)
Mohs excision	23 (28.0)
Unknown procedure	3 (3.7)
Surgical margins clear? (N = 64)	
Yes	53 (82.8)
No	11 (17.2)
Nodal evaluation (N = 81)	
None	17 (21.0)
maging only	24 (29.6)
SLNB only	18 (22.2)
Nodal dissection only	16 (19.8)
Both SLNB and ND	6 (7.4)
Adjuvant therapy (N = 82)	
Non-surgical	5 (6.1)
Surgery only	40 (48.8)
Surgery + radiation	37 (45.1)

### Endpoint analysis

**Fig 1:**
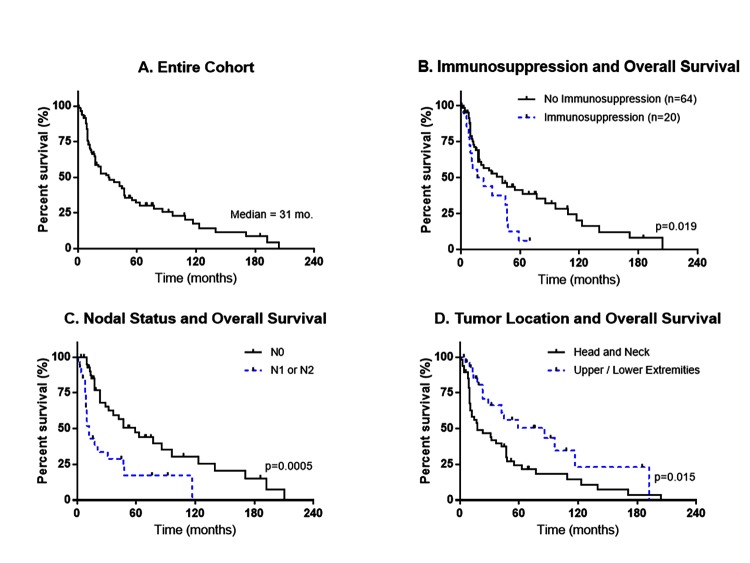
Kaplan-Meier graphs of overall survival and A) entire cohort; B) immunosuppression; C) nodal status; and D) primary tumor site.

The median time to follow-up for all patients, regardless of vital status, was 17 months (range 0–210 months). At the time of analysis, only 28 of 87 (32.2%) patients remained alive (Fig 1). Eleven of these patients have been lost to follow-up for more than one year. The 2-year and 5-year overall survival rates were 53.9% and 32.8% respectively while the median overall survival was 31 (95% CI: 18–47) months. Recurrences were confirmed for 27 (31.0%) patients; 23 (85.2%) with locoregional failure, 13 (48.1%) with distant metastases, and nine (33.3%) had both locoregional as well as distant metastases. Of note, locoregional recurrence occurred in 24% (12/50) patients with initial negative resection margin.

Univariate survival analyses were performed to evaluate the association of different patient and tumor characteristics with overall survival (Table 3). Immunosuppression status (P = 0.0190), COPD (P = 0.0045), site of primary tumor (P = 0.0098), N Stage (P = 0.0022), primary treatment (P = 0.0109) and Surgical Margins (P = 0.0063) were significantly associated with patient survival. Over the entire observation period, overall survival was significantly worse among immunosuppressed patients (hazard ratio, 2.01; 95% CI, 1.1–3.7), COPD patients (hazard ratio, 3.56; 95% CI, 1.38–9.2) and patients that received primary non-surgical treatment (hazard ratio, 4.4; 95% CI, 1.62–11.94) compared with surgical treatment as the referent group. Overall survival was significantly better among patients with primary tumor arising in the extremities (hazard ratio, 0.49; 95% CI, 0.27–0.89) compared with head and neck tumors as the referent group, N stage types pN0 (hazard ratio, 0.40; 95% CI, 0.18–0.90) and cN0 (hazard ratio, 0.34; 95% CI, 0.17–0.67) compared with N1/N2 as the referent group, and free surgical margins (hazard ratio, 0.38; 95% CI, 0.19–0.79). Variables for gender, smoking, CAD, DMII, previous cancer diagnosis and T Stage were not associated with overall survival in this cohort.

**Table 3:** Univariate Hazard Ratio Analysis.

Variable	Level	HR (95% CI)
Sex	M vs F	1.38 (0.8–2.36)
Smoking		1.03 (0.59–1.81)
Immunosuppression		2.01 (1.1–3.7) **
CAD		0.9 (0.49–1.63)
COPD		3.56 (1.38–9.2) **
Previous cancer		1.49 (0.87–2.57)
Site of Primary Tumor	H/N	REF
	Extremities	0.49 (0.27–0.89) **
	Other	2.57 (0.89–7.46) **
T stage	T1	REF
	T2/T3/T4	1.76 (0.91–3.4)
	T0	1.9 (0.44–8.22)
N stage	N1/N2	REF
	cN0	0.34 (0.17–0.67) **
	pN0	0.40 (0.18–0.90) ^†^ **
Treatment	Surgery only	REF
	Surgery + Adj. Radiation	1.10 (0.63–1.93)
	Non-Surgical	4.4 (1.62–11.94) **
Free surgical margins		0.38 (0.19–0.79) **
SLNB		0.56 (0.29–1.06)
ND		2.01 (1.11–3.63) **

** Statistically significant to p < 0.05

^†^ pN0 cohort did not have significantly better survival than the cN0 cohort (hazard ratio, 1.19; 95% CI, 0.53–2.68)

## Discussion

Our study is consistent with previous reports that factors such as nodal involvement and immunosuppression impact overall survival in patients with MCC. In addition, we show that patients with MCC arising on the head and neck, relative to those arising on the upper extremities, experience poorer overall survival (Fig 1). Patients with head and neck primaries also exhibit a trend towards increased nodal involvement at diagnosis suggesting that lymph node assessment at diagnosis or therapy targeting the draining lymphatic system may have an important role in this setting.

This cohort is comparable to previous epidemiological surveys and single-institution studies in terms of patient demographics [1, 2, 30, 32–36, 39, 40, 42, 46], being predominately composed of elderly white males. This similarity in patient population, particularly with larger population-based studies, may support the generalizability of our data. The median overall survival in this retrospective study (31 months) was indeed lower than many other single-institution studies, which tend to have median OS values of around 60–70 months, although the 5-year OS was within the published range of 30–60% [1, 26, 27, 32–34, 36, 39, 50]. As our institution is a tertiary care center, many patients present for a second opinion and remain in our system only during their treatment course. We did not exclude these patients from our study, but this inclusion lowered the follow-up length. This can be seen in the fact that the mean follow-up time is significantly longer than the median. Given these limitations, overall survival obtained with utilization of the social security death index represented the most meaningful and reliable endpoint to be derived from this patient population.

Our data support the current criteria for staging in place for MCC. The AJCC staging handbook guidelines include the size of the primary tumor, regional nodal basin status, and presence of distant metastases [48]. Involvement of lymph nodes (N1a, N1b, N2) was a poor prognostic factor compared to patients who were clinically or pathologically N0. This is consistent with a number of prior studies, which have demonstrated that patients with involved lymph nodes exhibit worse overall survival compared to those without nodal involvement (Table 4) [10, 30, 42, 43]. We did not observe an improvement in overall survival in patients with histologically confirmed node-negative staging. While we failed to observe a correlation between size of tumor and nodal involvement in this cohort, Iyer and colleagues demonstrated that the rate of lymph node involvement even for small (0.5 cm) tumors still approached 20% [40]. Taken together, these data suggest that nodal assessment should be performed for all patients with MCC and not be reserved for those with only advanced T stage disease. Since lymphadenectomy has been suggested to enhance disease-free survival [30, 51], it is important to identify patients who could benefit from this approach.

**Table 4:** Summary of current Merkel Cell Carcinoma literature.

Series (patient population)	Cases	Survival of Entire Cohort	Median F/U (months)	Effect of Nodal Eval	Effect of Adj. XRT
Morrison et al 1990 (MD Anderson)	54	30% (5-yr OS)	NR	NR	Prolonged DFS and OS
Meeuwissen et al 1995 (Queensland)	80	68% (3-yr OS)	NR	NR	Prolonged LC
Kokoska et al 1997 (St. Louis Univ.)	35	50% (2-yr OS)	31	ND showed improved OS (p<.01)	Prolonged OS (p = .03)
Ott et al 1999 (Mass. Gen. Hospital)	33	8 months (Median DFS)	37	NR	Prolonged LRC
Allen et al 2005 (Mem. Sloan Kettering)	251	64% (5-yr DSS)	40	pN0 showed improved DSS over cN0 (p = .009)	No effect on recurrence
Jabbour et al 2007 (Sydney)	82		23	NR	Prolonged both LC and OS (p = .033)
Kaae et al 2010 (Denmark)	185	45% (5-yr OS)	NR	NR	NR
Fields et al 2011 (Wash. U in St. Louis)	500	56% (5-yr OS)	36	NR	NR
Hui et al 2011 (Melbourne)	176		26	NR	Prolonged LRC
Tarantola et al 2013 (Mayo Clinic)	240	63% (5-yr OS)	40	No difference between (+/-) SLNB	Trend towards prolonged OS (p = 0.1)
Asgari et al 2014 (Kaiser Permanente Northern California)	218		29	pN0 showed improved DFS over cN0.	Prolonged LRC
Liang et al 2014 (Univ. of Wisconsin)	87	32% (5-yr OS)	17	pN0 showed no improved OS over cN0 (p = 0.61)*	No effect on OS (p = 0.32)
Mojica et al 2007 (SEER survey)	1665	49 months (Median OS)	40	NR	Prolonged OS
Lemos et al 2010 (National Cancer Database)	5823	40% (5-yr OS)	64	pN0 showed improved OS over cN0 (p<.0001)	NR
Kim et al 2013 (SEER survey)	747	NR	NR	NR	Prolonged OS (p = .03), no effect on DSS (p = .26)
Hasan et al 2013 (PUBMED Lit Review)	4475	NR	NR	NR	Prolonged OS (p<.001)

NR—not reported

* pN0 cohort did not have significantly better survival than the cN0 cohort (hazard ratio, 1.19; 95% CI, 0.53–2.68)

A key finding of this study is the difference in overall survival based on primary tumor location. Improved overall survival was observed in MCC patients with primaries localized to the extremities compared to those with head and neck primaries. The relatively poor survival of the head and neck patients may be attributable to a higher proportion of nodal involvement that may be related to the rich lymphatic system of the head and neck. Our data corroborates findings by Smith and colleagues who demonstrated increased nodal involvement for MCC of the head and neck when compared with primaries elsewhere [52]. Interestingly, they did not see a significant difference in disease-specific survival between head and neck and non-head and neck patients. This differs from our analysis in that primary MCCs of the trunk were included in their non-head and neck cohort; these exhibited poorer survival than those of the extremities. Omission of these could yield a difference between MCC of the head and neck and MCC of the extremities as demonstrated in our study. It is also possible that utilization of different endpoints (DSS vs. OS) was responsible for the apparent difference.

Development of MCC has previously been linked to chronic immunosuppression [10]. MCCs are more common among patients with HIV infection [53], CLL [54], and following solid organ transplant [54]. Past data have indicated that immunosuppression is also correlated with worse disease-free survival in MCC patients [34, 35]. Our data are consistent with these prior reports and suggest when possible immunosuppression should be minimized in an attempt to improve outcomes for immunosuppressed patients.

Adjuvant radiation, although not associated with an improved overall survival in our series, has been shown to be of use in many other studies [27–29, 31, 33–36, 38, 47]. One common limitation of assessing the role of adjuvant radiotherapy in a retrospective analysis is that significant selection biases are involved in the decision to both give and forego radiotherapy. It is possible that in an uncontrolled retrospective study, there may have been a bias towards administration of radiation in the cases of more advanced disease, explaining our findings. Our series did not include sufficient numbers of patients to compare the effect of radiotherapy within separate strata based upon staging, which may have identified a potential benefit to adjuvant radiotherapy. Over the last 5 years, a number of groups have reported their institutional results of patients with MCC (Table 4). A 2007 study of 1665 patients from National Cancer Institute SEER survey showed that the use of adjuvant radiation was associated with increased overall survival [31]. Another SEER study of 747 patients performed in 2013 showed improved OS but no change in disease specific survival [37]. Single institution studies have also shown increased locoregional control with adjuvant radiotherapy [26, 27, 29, 33, 35, 38, 50]. It is possible that our patient cohort could have had an increase in locoregional control associated with adjuvant radiation therapy. However, the number of recurrence events was limited, so meaningful conclusions could not be drawn the benefit of radiation treatment.

We acknowledge several limitations of this retrospective, single-institution study. Due to the referral patterns at our institution, a number of patients were seen only once during their disease course and limited data regarding the treatments they received was available. Several potential prognostic markers were likewise not routinely provided including p63, sarcomatous differentiation, and even merkel cell polyomavirus status are unable to be reported. Similarly, even in those patients in whom treatments were known, follow-up data regarding local control or development of metastases was poor thus limiting our ability to report on either local control or disease free survival. Both of these endpoints have been obtained in other studies and have proven to be useful, but here, these endpoints are not easily obtained due to the wide catchment area for our patients. However, given the very poor prognosis associated with recurrent MCC, the use of overall survival as our primary endpoint is a reasonable approach.

## Conclusions

This 30-year review at our institution reflects the aggressive nature and generally poor outcomes seen in patients with MCC. Patient history (immunosuppression) and initial presentation (head/neck location, nodal involvement) may provide prognostic cues to help guide clinicians in order to make more informed treatment decisions. As tumor size may not correlate with nodal involvement, it is important to fully stage MCC by evaluating the draining lymphatics. These data suggest that the importance of nodal evaluation is particularly relevant for patients with MCC arising on the head and neck, who have higher rates of lymph node involvement and worse outcomes. Clearly, additional studies are needed to continue to improve the care of patients with MCC.

## Supporting Information

S1 Table List of secondary malignancies by type. Some patients had multiple additional cancers.
(XLSX)
